# Genetic structure of the endangered Irrawaddy dolphin (*Orcaella brevirostris*) in the Gulf of Thailand

**DOI:** 10.1590/1678-4685-GMB-2020-0365

**Published:** 2021-04-02

**Authors:** Yufei Dai, Rachawadee Chantra, Kongkiat Kittiwattanawong, Liyuan Zhao, Watchara Sakornwimon, Reyilamu Aierken, Fuxing Wu, Xianyan Wang

**Affiliations:** 1 Third Institute of Oceanography, Ministry of Natural Resources, Laboratory of Marine Biology and Ecology, Xiamen, China.; 2 Fujian Provincial Key Laboratory of Marine Ecological Conservation and Restoration, Xiamen, China.; 3 Marine and Coastal Resources Research Center, The Upper Gulf of Thailand, Samut Sakhon, Thailand.; 4 Phuket Marine Biological Research Center, Phuket, Thailand.; 5 Marine and Coastal Resources Research Center, The Central Gulf of Thailand, Chumphon, Thailand.

**Keywords:** Mitochondrial DNA, microsatellite, cross-amplification, genetic differentiation, cetacean

## Abstract

The Irrawaddy dolphin (*Orcaella brevirostris*) is an endangered, small cetacean species which is widely distributed in rivers, estuaries, and coastal waters throughout the tropical and subtropical Indo-Pacific. Despite the extensive distribution of this species, little is known of individual movements or genetic exchange among regions in Thailand. Here, we evaluate the genetic diversity and genetic structure of *O. brevirostris* in the eastern, northern and western Gulf of Thailand, and Andaman Sea. Although phylogenetic relationships and network analysis based on 15 haplotypes obtained from 32 individuals reveal no obvious divergence, significant genetic differentiation in mitochondrial DNA (overall F_ST_ = 0.226, P < 0.001; Φ_ST_ = 0.252, P < 0.001) is apparent among regions. Of 18 tested microsatellite loci, 10 are polymorphic and successfully characterized in 28 individuals, revealing significant genetic differentiation (overall F_ST_ = 0.077, P < 0.05) among the four sampling sites. Structure analysis reveals two inferred genetic clusters. Additionally, Mantel analysis demonstrates individual-by-individual genetic distances and geographic distances follow an isolation-by-distance model. We speculate that the significant genetic structure of *O. brevirostris* in Thailand is associated with a combination of geographical distribution patterns, environmental and anthropogenic factors, and local adaptations.

## Introduction

The Irrawaddy dolphin (*Orcaella brevirostris*) is a small cetacean species which is widely distributed in rivers, estuaries, and coastal waters throughout the tropical and subtropical Indo-Pacific (Stacey and Arnold, 1999; Minton et al., 2013). Estuarine and coastal populations of this species occur from the northwestern Bay of Bengal, east through the Gulf of Thailand to the Philippines, and south to the Indonesian Archipelago (Stacey and Arnold, 1999; Krützen et al., 2018). Riverine subpopulations occur in three large rivers including Ayeyarwady in Myanmar, Mahakam in Indonesia, and Mekong in Cambodia and southern Lao People’s Democratic Republic (Smith and Hobbs, 2002; Kreb, 2004; Beasley et al., 2013). Other subpopulations occur in lagoons and marine appended lakes, such as Chilika in India, Songkhla in Thailand, and Malampaya Sound in the Philippines (Beasley *et al*., 2002; Dolar et al., 2002; Sutaria and Marsh, 2011).

Different habitat preferences are observed between the *O. brevirostris* populations in fresh waters and coastal waters. In rivers, *O. brevirostris* occur in deeper waters (10-50 m) at the confluences of two rivers, or above and below rapids, while in estuaries and coastal waters they frequent shallower depths (generally < 10 m) within a few kilometers of shore (Dolar et al., 2002; Smith and Hobbs, 2002; Smith *et al*., 2006; Minton et al., 2011; 2013; Jackson-Ricketts et al., 2018). Because of their patchy and fragmented distribution in rivers and coastal waters, Irrawaddy dolphins are particularly vulnerable to disturbance (Minton *et al*., 2017). This species is currently classified as Endangered by the International Union for Conservation of Nature (IUCN) in their Red List, and is listed in Appendix I of the Convention on International Trade in Endangered Species of Wild Flora and Fauna (CITES). Because of their small population sizes and declining number, and increasing anthropogenic threats, the three riverine subpopulations (Ayeyarwady, Mahakam, Mekong) and two lacustrine subpopulations (Songkhla, Malampaya) are classified as Critically Endangered by the IUCN (Minton *et al*., 2017).

In Thailand’s waters, *O. brevirostris* occur along the Andaman Sea coast, Songkhla Lake, and many locations along the coast surrounding the Gulf of Thailand (Chantrapornsyl et al., 1996; 1999; Anderson and Kinze, 1999; Beasley et al., 2002; Smith et al., 2003; Kittiwattanawong et al., 2007; Hines et al., 2015). Recently, field observations and acoustic studies targeting *O. brevirostris* were conducted at locations such as Trat Bay, Bangpakong Estuary, and Donsak, in the eastern, northern, and western Gulf of Thailand, respectively (Tongnunui et al., 2011; Hines *et al*., 2015; Jutapruet et al., 2017; Niu et al., 2019; Jackson-Ricketts et al., 2020). Additionally, significantly different isotope values in *O. brevirostris* teeth, indicating strong geographic variation and potential subpopulation structure in the Gulf of Thailand and Andaman Sea coast, have been reported (Jackson-Ricketts *et al*., 2018). Despite this, little is known about individual movements and genetic exchange of *O. brevirostris* among regions in Thailand.

Understanding genetic diversity and the genetic structure of populations is important to conserve and manage species, especially for inshore dolphins whose isolated populations are highly affected by human activities (Mace and Lande, 1991; Jefferson et al., 2009; Brown et al., 2014). Mitochondrial DNA (mtDNA) has been used as a genetic marker for reconstructing patterns of population demography, admixture, biogeography and speciation, because of its high mutation rate, maternal inheritance and lack of recombination, and high intracellular copy number (Wilson et al., 1985; Hurst and Jiggins, 2005). Recently, there are a few mtDNA-based studies on the genetic diversity and population structure of *O. brevirostris* throughout its distribution range. The mtDNA evidence shows that the Chilika population in India does not share any haplotypes with those of Thailand, Cambodia and Indonesia populations (Jayasankar et al., 2011). Another molecular genetic-related study suggests a long-standing isolation of the Mekong dolphin population from other Orcaella populations, with the remaining population now experiencing low genetic diversity (Krützen et al., 2018). Moreover, significant levels of genetic differentiation are found among three *O. brevirostris* populations, Chilika Lagoon in India, the eastern Gulf of Thailand, and the Mekong River in Cambodia, indicating strong genetic differentiation between coastal and riverine populations and among different geographic locations (Caballero et al., 2019).

Microsatellite markers are short tandem repeats of 1-6 nucleotides that are widespread in the nuclear genomes of most taxa. Due to their high mutation rates, microsatellites are also considered to be robust and informative DNA markers for solving ecological and molecular genetic-related questions, such as those pertaining to bottlenecks, kinship, population structure, and migration (Selkoe and Toonen, 2006; De Barba et al., 2017). There are few known microsatellite sequences for *O. brevirostris*, although several molecular genetic-related studies have been undertaken on this species (Jayasankar et al., 2011; Krützen et al., 2018; Caballero et al., 2019; Kundu et al., 2019). Fortunately, microsatellites with highly conserved flanking regions often allow cross-species amplification from congeneric or confamilial taxa, especially for vertebrates, including mammals (Selkoe and Toonen, 2006; Barbara et al., 2007).

Our research aims were to: (1) assess transferability and polymorphism of microsatellite markers for *O. brevirostris* from closely related dolphin species; (2) evaluate genetic diversity of *O. brevirostris* using samples obtained from throughout the Gulf of Thailand and Andaman Sea coast using mitochondrial control region sequences and nuclear microsatellite markers; and (3) estimate levels of genetic differentiation among these *O. brevirostris* individuals in Thailand for both marker types.

## Material and Methods

**Sampling**

All fieldwork was under permits from the Ministry of Agriculture and Rural Affairs of China, and with approval from the Department of Marine and Coastal Resources of Thailand. The relevant CITES Permits (No. 2018CN/IC000475/CH) were obtained for import of samples. There was no issue on ethics in this study.

### Sample collection and DNA extraction

**Figure 1 -:**
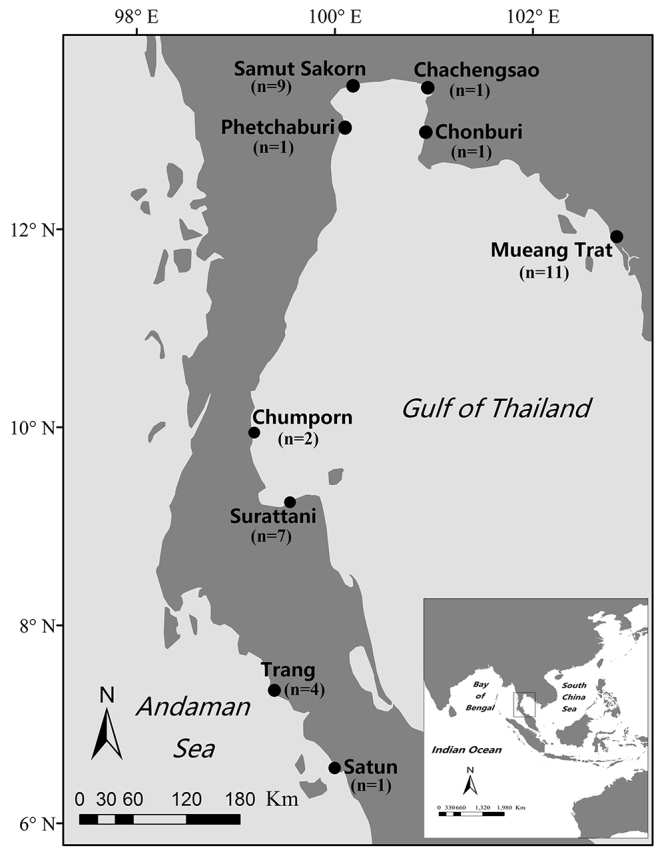
Locations of sample collection in the Gulf of Thailand and Andaman Sea sites. Mueang Trat represents the eastern Gulf of Thailand (EG); Chonburi, Chachengsao, Samut Sakorn, and Phetchaburi make up the northern Gulf (NG); Chumporn and Surattani make up the western Gulf (WG); Trang and Satun represent the Andaman Sea site (AS).

From 2010 to 2018, muscle or skin samples were collected from 37 dead (stranded) *O. brevirostris* individuals recovered from nine sites in Thailand: Chachengsao (n = 1), Chonburi (n = 1), Chumporn (n = 2), Mueang Trat (n = 11), Phetchaburi (n = 1), Samut Sakorn (n = 9), Satun (n = 1), Surattani (n = 7), and Trang (n = 4). Geographic coordinates for all except one individual from Phetchaburi were recorded at the time of collection. Samples were grouped into four general regions as described in Jackson-Ricketts et al. (2018), each region encompassing specific sites: (1) Mueang Trat, representing the eastern Gulf of Thailand (EG, n = 11), (2) Chonburi, Chachengsao, Samut Sakorn, and Phetchaburi, making up the northern Gulf (NG, n = 12), (3) Chumporn and Surattani, making up the western Gulf (WG, n = 9), and (4) Trang and Satun, representing Andaman Sea sites (AS, n = 5) (Figure 1). Genomic DNA from minced tissue samples was extracted using DNeasy blood and tissue extraction kits (QIAGEN, Valencia, USA) according to the manufacturer instructions.

### Mitochondrial DNA amplification

A fragment of the mitochondrial control region was amplified using forward primer Ce-CRF 5′-GAATTCCCCGGTCTTGTAAACC-3′ and reverse primer Ce-CRR 5′-TCTCGAGATTTTCAGTGTCTTGCTTT-3′ (Hoelzel et al., 1991). Total PCR volume of (50 μL) contained approximately 50 ng of genomic DNA, 1×Easy Taq Mix (Takara, Dalian, China), 1 μL of each forward and reverse primer, and ddH_2_O to make up the final volume. The PCR profile comprised an initial denaturation step at 94 ℃ for 3 min, 35 cycles of 94 ℃ for 30 s, annealing at 50 ℃ for 1min, extension at 72 ℃ for 1min, and a final extension at 72 ℃ for 10 min. All PCR products were sequenced in both directions on an ABI 3730 automated DNA sequencer (Applied Biosystems).

### Microsatellites crossamplification

We selected a total of 18 microsatellite markers (Table S1, data unpublished), initially isolated from the Indo-Pacific humpback dolphin *Sousa chinensis*, for this study. These loci were all ‘perfect-type’ and long tandem repeat motifs (e.g., tetra- or pentanucleotide), and demonstrated high polymorphism in Thailand *S. chinensis* populations (data unpublished). Microsatellites were allocated into 6 multiplex PCR panels using software MPprimer (Shen et al., 2010), based on annealing temperature, complementarity of primer pairs, and allele size range. The 5’ end of each forward primer was labeled with a fluorescent dye (6-FAM, VIC or NED). The total PCR volume (20 μL) consisted of approximately 50 ng of genomic DNA, 1×Multiplex PCR Kit (Takara, Dalian, China), the optimal dosage (Table S1) of each forward and reverse primer, and ddH_2_O added to make up the final volume. PCR conditions involved an initial denaturation step at 94 °C for 3 min, followed by 32 cycles of 94 °C for 30 s, the specific annealing temperature (Table S1) for 90 s, extension at 72 °C for 60 s, and a final extension for 30 min at 60 °C. PCR products were run on an ABI 3730XL automated DNA sequencer (Applied Biosystems) using GeneScan LIZ 500 as the internal size standard. Allele sizes were automatically scored with GeneMapper version 4.1 (Applied Biosystems) and manually checked.

### Detecting genotyping errors

Genomic DNA obtained from dead individuals may be low in quantity and/or quality, be at increased risk of contamination, and more susceptible to genotyping errors such as allele dropouts and false alleles (Taberlet *et al*., 1996; Bonin et al., 2004; Pompanon et al., 2005). Therefore, in each multiple PCR batch we used one individual as a positive control for genotyping each locus separately to ensure consistent amplification of alleles. A negative control with no DNA template was also used in each PCR batch to detect possible of contamination during PCR amplification. Moreover, we randomly retested genotypes from four individuals (> 10 %) to estimate genotyping error rates for across all 18 loci.

### Statistical analyses

Mitochondrial DNA data analysis

Raw mtDNA sequences were aligned using the Clustal-W algorithm implemented in the program MEGA version 5.0 (Tamura et al., 2011), then manually checked and edited. MEGA was also used to calculate nucleotide composition, and variable and conserved sites. Diversity measures including numbers of haplotypes (H), specific haplotypes (SH), and nucleotide (Hd) and haplotype diversities (π) for each region, were analyzed using DnaSP version 5 (Librado and Rozas, 2009). Phylogenetic relationships among haplotypes were constructed using the neighbor-joining (NJ) method implemented with 1000 bootstrap replicates in MEGA. A haplotype network was constructed using the software PopART (Leigh and Bryant, 2015). We also used Arlequin version 3.0 (Excoffier et al., 2005) to investigate genetic variation among regions by calculating both F_ST_ and Φ_ST_ values with 1000 random permutations.

Microsatellite data analysis

GenAIEx version 6.501 (Peakall and Smouse 2006, 2012) was used to estimate the number of alleles per locus (Na), effective number of alleles (Ne), observed heterozygosity (Ho), expected heterozygosity (He), unbiased expected heterozygosity (uHe), Shannon′s information index (I), and Fixation Index (F). Genepop version 4.0.7 (Rousset, 2008) was used to test departure from Hardy-Weinberg equilibrium (HWE) and linkage disequilibrium (LD) among all pairs of loci. Myriads version 1.1 (Carvajal-Rodriguez, 2018) was performed to correct the p-value-based multiple testing by Bonferroni sequential correction procedures. To check for signs of genetic bottlenecks, which cause an heterozygosity excess in populations, BOTTLENECK version 1.2.02 (Cornuet and Luikart, 1996; Piry et al., 1999) was used under the infinite allele model (IAM), stepwise mutation model (SMM), and two-phased model (TPM), based on 1000 iterations. We used the Wilcoxon sign-rank test to estimate significance because only 10 microsatellite loci were included in analysis (Wilcoxon, 1945). Micro-Checker version 2.2.3 (Van Oosterhout et al., 2004) was used to detect occurrences of null alleles, allele dropout, or scoring error for each locus, with 95 % confidence intervals.

Genetic structure and number of genetic clusters (K) were determined using Structure 2.3.4 (Pritchard et al., 2000) based on genotyping data generated from the 6 multiplex PCR panels in this study. For all analyses, the length of the burn-in period was set to 10^5^ iterations, followed by 10^6^ in the number of MCMC repetitions. Then, the Admixture model was used with correlated allele frequencies. The Locprior model was chosen to infer possible weak population structure with the assistance of sample group information (Hubisz et al., 2009). The number of inferred K was set between 1 and 6, and 20 independent replicates were run for each K value. Subsequently, the web program Structure Harvester version 0.6.94 (Earl and VonHoldt, 2012) was used to calculate the Delta K value and determine the best number of K clusters (Evanno et al., 2005). We used CLUMPP version 1.1.2 (Jakobsson and Rosenberg, 2007) to summarize the optimal alignment of the 20 replicates for the same K value. Final results were displayed graphically with Distruct version 1.1 (Rosenberg, 2004).

FSTAT version 2.9.3.2 (Goudet, 1995, update in Feb. 2002) was used to estimate genetic differentiation F_ST_ (Weir and Cockerham, 1984) among the four geographic regions based on 1000 permutations. A principal coordinate analysis (PCoA) was performed in GenAIEx based on the standardized covariance of the individual-by-individual genetic distance matrix (Peakall et al., 1995; Smouse and Peakall, 1999). In addition, Mantel analysis (Mantel, 1967) was used in GenAIEx to test isolation by distance (IBD) by performing the correlation between matrices of individual-by-individual genetic distances and geographic distances measured as the distance between two individuals calculated from sampling location coordinates. We conducted a second IBD analysis excluding the genetic and geographical data for AS samples because of the large distance between AS and other regions. Both tests were run with 999 random permutations in GenAIEx.

### Data availability

The 18 tested microsatellite sequences and 32 obtained mtDNA control region sequences (including 15 mtDNA haplotypes) reported in this paper have been deposited into the GenBank database under the accession numbers of MK766845-MK766870 (Table S1) and MT738330-MT738361 (Table S2), respectively.

## Results

### Available genetic data for analysis

No contamination was detected during PCRs, and no genotyping errors were observed when randomly retesting four individuals. Among the 18 loci tested, two (Sch5878 and Sch5685) were excluded because of unsuccessful amplification, and six (Sch7427, Sch193, Sch4657, Sch5373, Sch974, Sch5094) were excluded because they were monomorphic. The remaining 10 loci were polymorphic in the four sampling locations.

**Figure 2 -:**
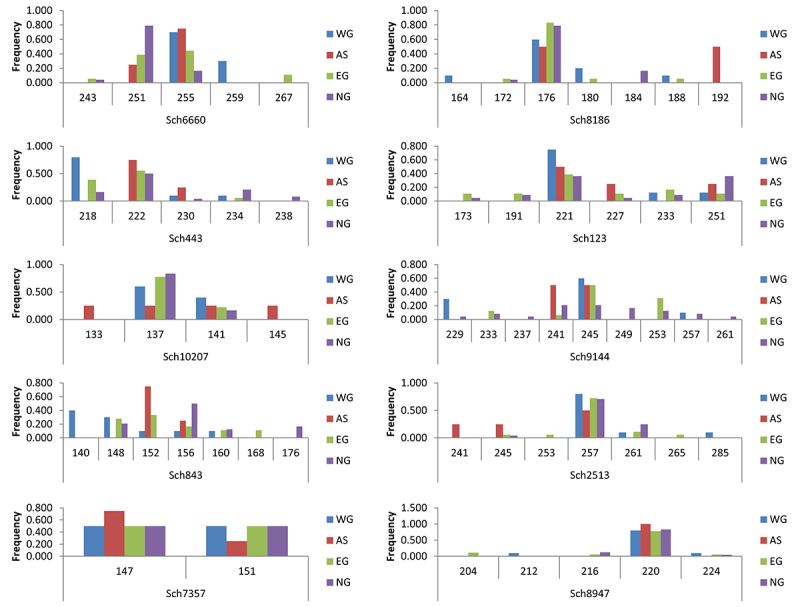
Allele frequencies with graphs by each sampling location and each locus for microsatellite data. Allele frequencies are displayed for 10 polymorphic microsatellites, and different colors represent different regions in Thailand.

Due to the low quality of some DNA samples, we obtained mtDNA sequences for 32 individuals (Table S2) only, including two from AS, eight from WG, 10 from EG, and 12 from NG. For microsatellite data analysis, only DNA samples for which at least eight of the 10 polymorphic loci were successfully genotyped were included. Therefore, nine samples (two from EG, three from AS, and four from WG) were removed because of poor amplification success. Finally, we generated genotypes of 10 microsatellites for 28 individuals only, including two individuals from AS, five from WG, nine from EG, and 12 from NG. Allele frequency distributions in the four geographic regions based on 10 polymorphic loci are graphically represented in Figure 2.

### Genetic diversity

**Figure 3 -:**
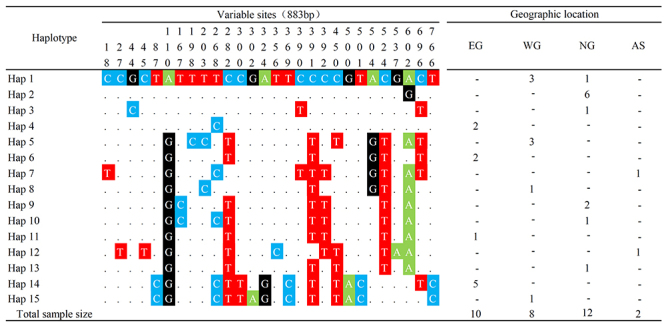
Polymorphic sites of mtDNA haplotypes identified among the four geographic regions in Thailand. Nucleotide position and geographic location are at the top, respectively.

Based on mtDNA data, 15 haplotypes were found in the 32 individuals by a consensus 883 bp sequences from the four geographic locations (Figure 3). Only one haplotype was shared between NG and WG. Genetic diversity estimates of H, SH, Hd, and π values for each region are summarized in Table 1. NG showed the most haplotypes (n = 6), while AS exhibited the least (n = 2). Estimated Hd was high (mean value = 0.925 ± 0.025), but observed π was relatively low (mean 0.009 ± 0.001). Although AS exhibited the highest Hd and π values among the four geographic regions, only 2 individuals were included in genetic analysis.

**Table 1 -:** Information and molecular indices for *O. brevirostris* based on mtDNA control region sequences.

Population	Number	H	SH	Hd ± SD	π ± SD
WG	8	4	3	0.786 +/- 0.113	0.009 +/-0.002
AS	2	2	2	1.000 +/- 0.500	0.014 +/- 0.007
EG	10	4	4	0.733 +/- 0.120	0.009 +/- 0.001
NG	12	6	5	0.758 +/- 0.122	0.005 +/- 0.001
Total	32	15	14	0.925 +/- 0.025	0.009 +/- 0.001

Note: H is the number of haplotypes, SH is the number of specific haplotypes, Hd is the haplotype diversity, π is the nucleotide diversity.

**Figure 4 -:**
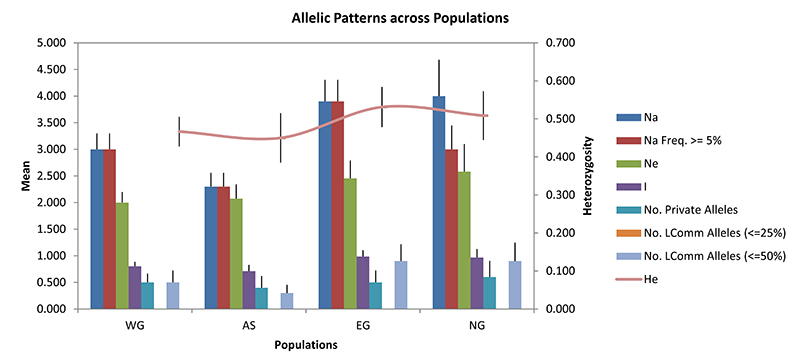
Allelic patterns across regions in the geographic distribution of *O. brevirostris* in Thailand. Na is the number of different alleles, Na Freq. is the number of common alleles with a frequency ≥ 5 %, Ne is the effective number of alleles, I is the Shannon’s information index, No. LComm Alleles are the number of locally common alleles (Freq. >= 5 %) found in 50 % or 25 % or fewer populations, and He represented by the curve is the expected heterozygosity.

Based on microsatellite DNA data, genetic diversity estimates of Na, Ne, Ho, He, uHe, I and F values for each sampling site are summarized in Table 2. Average Na and Ne were highest in NG, with values of 4.000 ± 0.683 and 2.581 ± 0.519, respectively. The highest mean He and I were in EG, with values 0.531 ± 0.053 and 0.986 ± 0.117, respectively (Figure 4). Although AS had the lowest I (0.710 ± 0.119) and He (0.450 ± 0.065), it had the highest Ho (0.600 ± 0.125) and uHe (0.633 ± 0.096). One locus significantly deviated from HWE (P < 0.05) in each of WG, EG, and NG (Table S3). Except for AS (because the sample size was too small to analyze), the other three locations had no significant bottleneck under any model of mutation (Wilcoxon test: P > 0.05). No significant LD was found in any pairs of the 10 tested loci after Bonferroni sequential correction. No evidence of large allele dropout or scoring errors was detected by Micro-Checker.

**Table 2 -:** Genetic diversity parameters of *O. brevirostris* in the four geographic regions in Thailand.

Sampling region		N	Na	Ne	I	Ho	He	uHe	F
WG	Mean	4.900	3.000	2.000	0.804	0.530	0.467	0.520	-0.102
(n = 5)	S.E.	0.100	0.298	0.198	0.083	0.084	0.039	0.043	0.120
AS	Mean	1.900	2.300	2.073	0.710	0.600	0.450	0.633	-0.319
(n = 2)	S.E.	0.100	0.260	0.268	0.119	0.125	0.065	0.096	0.172
EG	Mean	8.900	3.900	2.456	0.986	0.507	0.531	0.563	0.064
(n = 9)	S.E.	0.100	0.407	0.334	0.117	0.066	0.053	0.056	0.070
NG	Mean	11.900	4.000	2.581	0.966	0.522	0.509	0.531	-0.026
(n = 12)	S.E.	0.100	0.683	0.519	0.162	0.071	0.064	0.067	0.050

Note: N is the number of sample size, Na is the number of alleles, Ne is the number of effective alleles, I is the Shannon′s information index, Ho is the observed heterozygosity, He is the expected heterozygosity, uHe is the unbiased expected heterozygosity, F is the fixation index, S.E. is the standard error.

**Table 3 -:** Matrix of pairwise Φ_ST_ (above diagonal) and F_ST_ (below diagonal) values among the four geographic regions based on mtDNA control region sequences.

Φ_ST_	WG	AS	EG	NG
F_ST_				
WG	-	0.118	0.197*	0.160*
AS	0.158	-	0.291*	0.363*
EG	0.242**	0.202	-	0.348**
NG	0.205**	0.184	0.254**	-

Note: * P < 0.05, ** P < 0.01.

ANOVA results revealed Na values differed significantly from each other among sampled regions (ANOVA: F_3, 36_ = 3.279, P = 0.032), but Ne values did not (F_3, 36_ = 0.657, P = 0.584). There were no significant differences among He (F_3, 36_ = 0.442, P = 0.725) and uHe (F_3, 36_ = 0.559, P = 0.646) values. Additionally, no significant differences were found among Ho (F_3, 36_ = 0.213, P = 0.887) and I (F_3, 36_ = 1.151, P = 0.342) values from any of the four geographic regions.

### Genetic differentiation

**Figure 5 -:**
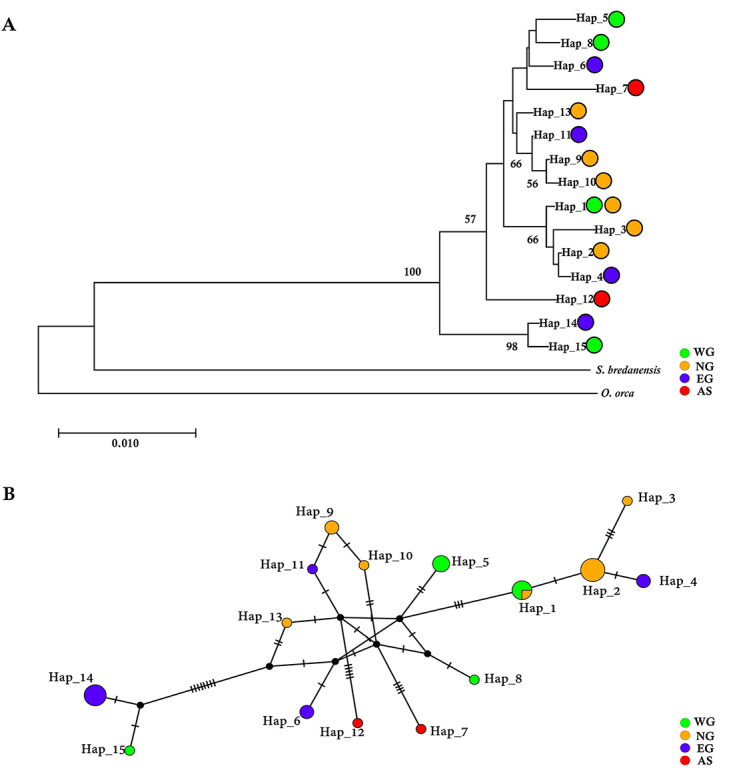
Genetic structures of all 15 mtDNA haplotypes of *O. brevirostris* in Thailand. (A) Neighbour-joining tree of mtDNA haplotypes. *Orcinus orca* and *Steno bredanensis* are used as the outgroup species. Only bootstraps values > 50 % are shown. (B) Network of mtDNA haplotypes. Size of the circles represents the frequency of haplotype, and dash lines represent the number of mutational steps between haplotypes. Colors indicate the sampling location(s) for each haplotype.

The NJ tree was constructed based on the 15 haplotypes of *O. brevirostris* and the two outgroup mtDNA sequences from *Orcinus orca* and *Steno bredanensis*. Haplotypes from different regions were randomly distributed on the NJ tree, with no phylogenetic structure corresponding to geography apparent (Figure 5A). The network also showed similar results with the NJ tree, without obvious divergence of haplotypes from different sites. Levels of divergence among most haplotypes were low. However, divergence from Hap_14 in EG and Hap_15 in WG to other haplotypes was extremely high. Both Hap_7 and Hap_12 (defined by two AS individuals) were also highly divergent from other haplotypes (Figure 5B).

AMOVA results revealed significant high genetic differentiation in mtDNA (overall F_ST_ = 0.226, P < 0.001; Φ_ST_ = 0.252, P < 0.001) among Thailand *O. brevirostris* individuals. Pairwise F_ST_ and Φ_ST_ values revealed moderate to high levels of genetic differentiation between different region pairs, but some cases showed no significant differentiation between AS and other region pairs (Table 3). For microsatellites, the estimated F_ST_ value revealed low but significant genetic differentiation (overall F_ST_ = 0.077, P < 0.05) among the four geographic regions. Low to moderate levels of genetic differentiation were detected between different region pairs. Estimates of pairwise F_ST_ are presented in Table 4. Pairwise differentiations were all significant (P < 0.05), except for the pair of EG and AS, which had the lowest genetic differentiation (pairwise F_ST_ = 0.008, P > 0.05). Some pairwise F_ST_ values were moderately differentiated, with the highest genetic differentiation found between WG and NG (pairwise F_ST_ = 0.166, P < 0.01). AMOVA results for the degree of variance in *O. brevirostris* individuals are summarized in Table 5. There was 7.706 % genetic variance among the four geographic regions, 6.676 % variance among individuals within region, and 85.618 % variance within individuals.

**Table 4 -:** Pairwise F_ST_ estimates among the four geographic regions based on microsatellites.

Population	WG	AS	EG	NG
WG	-			
AS	0.121*	-		
EG	0.060*	0.008	-	
NG	0.166**	0.101*	0.034*	-

Note: * P < 0.05, ** P < 0.01.

**Table 5 -:** Analysis of molecular variance of *O. brevirostris* in the four sampled regions.

Source of variation	df	SS	MS	Est. Var.	%
Among populations	3	17.679	5.893	0.231	7.706
Among individuals with population	24	71.339	2.972	0.201	6.676
Within individuals	28	72.000	2.571	2.571	85.618
Total	55	161.018		3.003	100

Note: df is the degrees of freedom, SS is the sums of squares, MS is the mean squares, Est. Var. is the estimated variance within and among populations.

**Figure 6 -:**
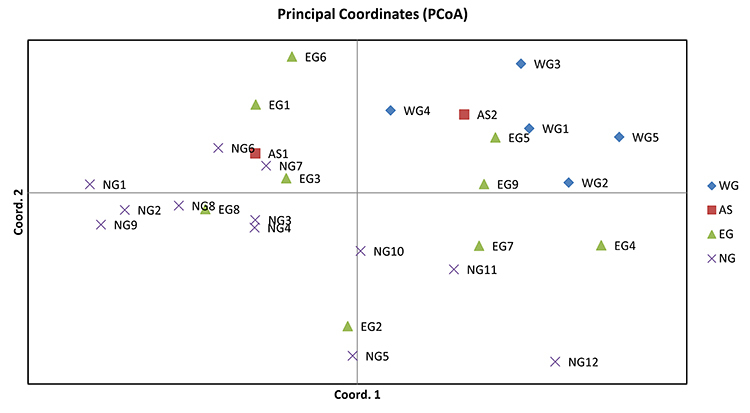
Principal coordinate analysis plots of individuals based on the standardized covariance of genetic distance matrix. Different shapes and colors represent different geographic regions in Thailand. Axis coordinates 1 and 2 account for 17.901 % and 12.988 % of the total variance, respectively.

PCoA plots of individuals based on the standardized covariance of genetic distance matrix revealed that the large genetic distance was detected between individuals from WG and NG, indicating the high level of genetic differentiation. Axis coordinates 1 and 2 accounted for 17.901 % and 12.988 % of the total variance, respectively (Figure 6).

**Figure 7 -:**
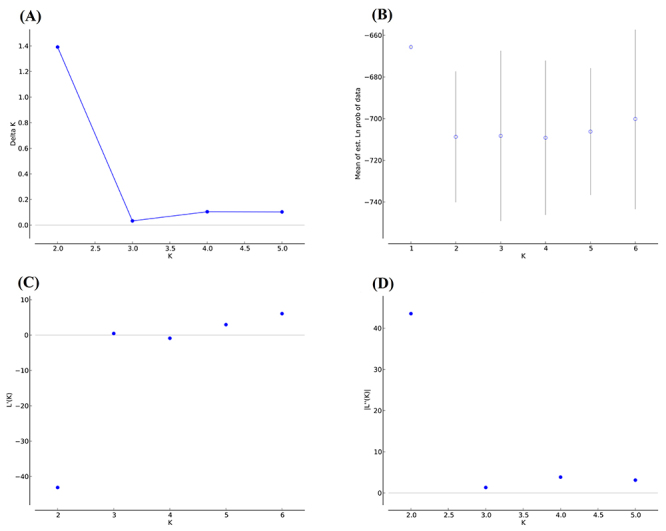
Optimal K value determined by Structure Harvester online program. (A) Delta K; (B) L (K) (mean ± SD); (C) Rate of change of the likelihood distribution; and (D) Absolute value of the 2nd order rate of change of the likelihood distribution.

**Figure 8 -:**
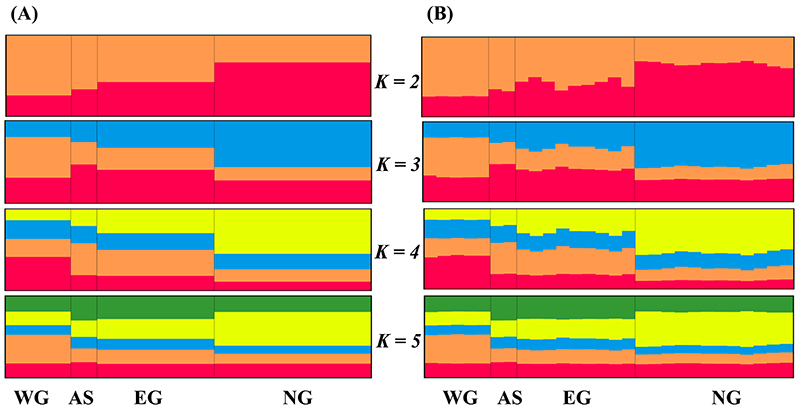
Results of clustering (K varied from 2 to 5) calculated by Structure program based on (A) the population Q-matrix, and (B) the individual Q-matrix. Black vertical lines separate the four geographic regions in Thailand, and different colors represent the possible genetic clusters.

Structure Harvester analysis revealed there were most likely only two clusters (Delta K = 2.0, Figure 7). The modal value of Delta K can best show the real number of groups, based on the second order rate of change with respect to K of the likelihood function (Evanno et al., 2005). However, the graphical output by Distruct indicated that all sampled individuals exhibited admixture in the possible genetic clusters, with K ranging 2 to 5 (Figure 8). Even at K = 2, a weak genetic structure for the two inferred genetic clusters remained apparent. Cluster I was predominant in three locations (WG, AS, and EG), with assignment probabilities were 74.5 %, 66.9 %, and 57.9 %, respectively. Cluster II was predominant in NG, with an assignment probability of 66.7 %. We also tried to identify possible partitions within cluster I and cluster II, but none was apparent.

**Figure 9 -:**
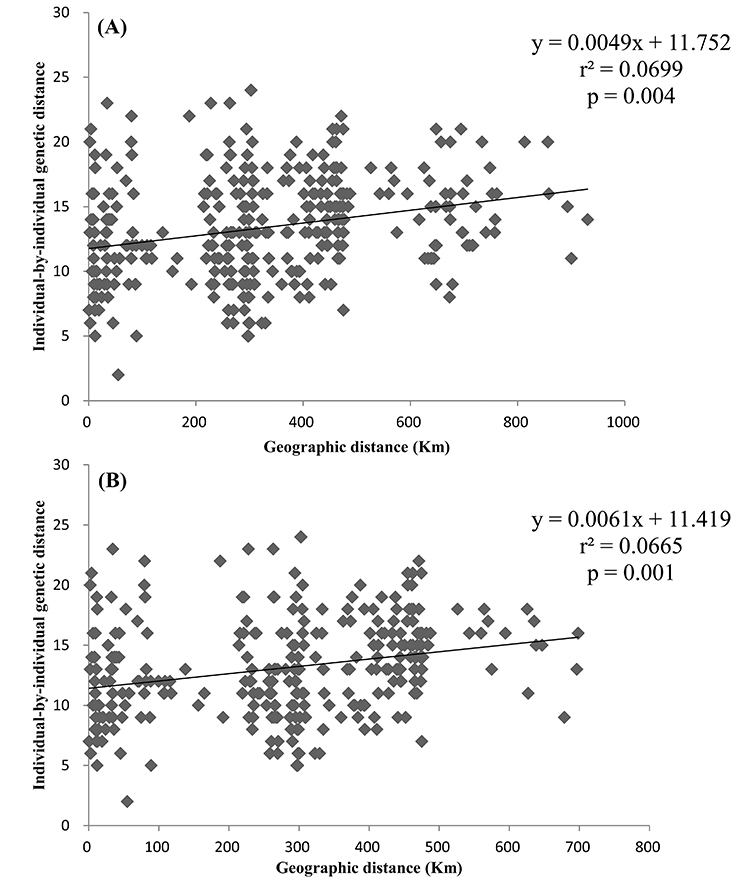
Isolation by distance plots using individual-by-individual genetic distances and geographic distances (km) among (A) the four (WG, AS, EG, and NG), and (B) the three (WG, EG, and NG) sampling sites in Thailand. Geographic and microsatellite data of 27 individuals were included in analysis because of the missing location coordinates of one individual in Phetchaburi (NG).

Mantel tests revealed a positive and weak correlation (r^2^ = 0.0699, P = 0.004) between the individual-by-individual genetic distances and geographic distances, indicating a pattern of IBD among the four geographic regions (Figure 9A). The result remained positive and significant when geographic coordinates and microsatellite data for two AS individuals were removed (r^2^ = 0.0665, P = 0.001; Figure 9B).

## Discussion

Some molecular markers with highly conserved primers have strong versatility during PCR. They can be invariant within species and sometimes even across broad taxonomic groups (Selkoe and Toonen, 2006). Microsatellites with highly conserved primer regions may be transferred from closely related species, but they rarely work across broad taxonomic groups (Glenn and Schable, 2005). Ten loci cross-amplified from other cetaceans (including *Tursiops aduncus*, *T. truncatus*, and *O. orca*) proved polymorphic in Mekong River *O. brevirostris* (Krützen et al., 2018). We report 10 of 18 tested loci isolated from *S. chinensis* to be polymorphic among *O. brevirostris* individuals from the four geographic locations in Thailand. These polymorphic markers will be useful for future molecular genetics studies on *O. brevirostris* and related species.

Based on microsatellite data, levels of genetic diversity we detected for *O. brevirostris* in Thailand waters (combined Na = 5.90, Ne = 2.87, uHe = 0.59, and Ho = 0.51) are higher than those from Mekong River (combined Na = 3.40, Ne = 1.91, uHe = 0.45, and Ho = 0.41) reported by Krützen et al. (2018). Differences in genetic diversity among *O. brevirostris* populations may be associated with the different polymorphic microsatellite markers used in these two studies. However, our analysis of mtDNA data also reveals that the haplotype and nucleotide diversities of *O. brevirostris* in Thailand (Hd = 0.925, π = 0.900 %) are much higher than those in Mekong River (Hd = 0.418, π = 0.120 %) and Chilika Lagoon (Hd = 0.345, π = 0.078 %) reported by Caballero et al. (2019). Even if we remove the two individuals from AS, the genetic diversity of *O. brevirostris* within the Gulf of Thailand remains high (Hd = 0.915 and π = 0.860 %), which is comparable to those in the Gulf of Thailand (Hd = 0.845 and π = 1.150 %) reported by Caballero *et al*. (2019). A similar situation is reported for the similarly small Indo-Pacific finless porpoise *Neophocaena phocaenoides*, for which the genetic diversity of a riverine population was lower than coastal populations (Jia et al., 2014). It is likely that decreased genetic diversity in *O. brevirostris* in Mekong River and Chilika Lagoon is related to a small effective population size and long-term isolation.

ANOVA based on microsatellite data reveals significantly different Na values among the four sampling sites in Thailand, with the mean Na in AS the lowest. However, Ne values from these locations did not significantly differ. Ne is an estimate of the number of equally frequent alleles in an ideal population, which enables meaningful comparisons of allele diversity across loci with diverse allele frequency distributions (Peakall and Smouse, 2012, Appendix 1). There is also no significant difference in other genetic diversity estimators, such as mean values for He, uHe, Ho and I values. Compared with He and uHe, the I value may be a better measure of allelic and genetic diversity, because it is not bounded by 1 (Peakall and Smouse, 2012, Appendix 1). Additionally, analysis of mtDNA data reveals Hd and π values in AS are much higher than those in the other three regions. Results of genetic diversity indices in AS may be controversial because we could include only two individuals in genetic analysis. No significant differences were found in genetic diversity among WG, EG, and NG in the Gulf of Thailand.

Overall, genetic differentiation among geographic regions was statistically significant based on mtDNA and microsatellite analyses. Our results are consistent with statistical differences in average stable isotope values reported from *O. brevirostris* teeth in the same regions by Jackson-Ricketts et al. (2018), indicating distinct geographic groups (Eastern Gulf, Northern Gulf, Western Gulf, and Andaman Sea) may exist in Thailand’s waters. However, no significant difference was detected for average tooth stable isotope values between northern and western Gulf regions. We report, to the contrary, significant genetic differences between NG and WG, suggesting *O. brevirostris* in these two regions are unlikely to be components of one large subpopulation, as stable isotope analysis is able to detect ecological populations, which may not correspond to genetic populations. Therefore, a lack of observed isotope differences might be due to similarities in habitat biochemistry in these two regions.

Our microsatellite data reveals the genetic structure of *O. brevirostris* in Thailand follows an IBD model, suggesting individual movements and exchanges are more likely to occur between adjacent regions. Therefore, the significant genetic differentiation detected between WG and NG regions might be explained by geographic distance (the mean distance between WG and NG individuals is 494.481 km), which is larger than individual dispersal distances. Although smaller geographic distances (mean 228.072 km) between EG and NG, significant genetic differentiation (both mtDNA and microsatellite data) remained between them. No significant genetic differentiation was found between AS and other regions based on different types of molecular markers, despite considerable distances between them (mean 653.853 km) and obvious environmental breaks, such as the Strait of Malacca. Perhaps this is an artefact of small sample sizes in AS, and that more genetic data are needed to accurately determine population structure between this region and elsewhere.

Environmental factors and coastal development have likely played important roles in driving genetic differentiation of *O. brevirostris* in Thailand. Geographical barriers formed from oceanographic variables, such as ocean currents, upwelling, bathymetry, sea surface temperature, primary productivity, and salinity, can affect cetacean genetic structure (Bilgmann et al., 2007; Mendez et al., 2010; 2011; Amaral et al., 2012; 2017). In Thailand, *O. brevirostris* have been reported from along the coast of Trat province and the Bang Pakong Estuary adjacent to the Chonburi province (Tongnunui et al., 2011; Hines et al., 2015; Jackson-Ricketts et al., 2020). However, *O. brevirostris* today appears to be absent from approximately 250 km of coastline neighboring Chanthaburi and Rayong provinces, between Chonburi and Trat provinces. Shallow depths, high water turbidity, and short distances to river mouths may be linked to the distribution of *O. brevirostris* in the northern and eastern Gulf of Thailand (Tongnunui *et al*., 2011; Jackson-Ricketts *et al*., 2020). Anthropogenic barriers to individual dispersal caused by coastal development may also contribute to population fragmentation of inshore dolphins (Brown et al., 2014). Given anecdotal historical evidence for *O. brevirostris* occurring in Chanthaburi, its absence today may be also be related to higher levels of fishing pressure and industrial development in Chanthaburi and Rayong provinces (Jackson-Ricketts *et al*., 2020).

In addition to geographic distribution patterns, and environmental and anthropogenic factors, adaptations caused by the complex social behaviors of cetaceans may contribute to genetic divergence (Mendez et al., 2013; Fruet et al., 2014). Some social and specialized foraging behaviors have been reported to be associated with significant levels of population structure in *T. aduncus* (Krützen et al., 2004; Frère et al., 2010) and *O. orca* (Ford et al., 1998; Matkin et al., 2007; Riesch et al., 2012). We speculate that local adaptations related to foraging strategies may have contributed to genetic differentiation of *O. brevirostris* in Thailand, because isotopic dietary analysis reveals *O. brevirostris* forages on different proportions of prey in neighboring locations where geographic barriers in the eastern Gulf of Thailand are lacking (Jackson-Ricketts et al., 2018). Therefore, *O. brevirostris* in Thailand may have high site fidelity because of local prey resources and specialized foraging behavior.

## Conclusion

In this study, 10 microsatellites were successfully cross-amplified and demonstrated to be polymorphic in *O. brevirostris* from the eastern, western, and northern Gulf of Thailand and Andaman Sea coast. Based on analyses of both mtDNA and microsatellites, genetic diversity does not differ significantly among sampling locations in the Gulf of Thailand, but significant genetic differentiation is apparent between different region pairs. We speculate that significant genetic structure is associated with a combination of geographical distribution patterns, environmental and anthropogenic factors, and local behavioral adaptations.

Caution interpreting these results should be exercised because our sample size for each region was small, and samples were collected in an opportunistic manner, in that all individuals were dead (beach stranded). Our results, together with isotope variability in teeth (Jackson-Ricketts et al., 2018), indicate that *O. brevirostris* may use specific habitats and have restricted home ranges throughout the life cycle in Thailand’s waters. An improved understanding of the level of conservation and management for *O. brevirostris* in Thailand’s waters will require more attention being paid to demographic, ecological and genetic-related issues regarding this endangered species.

## Supplementary Material

The following online material is available for this article:

Table S1 - Multiplex design information for all the tested 18 microsatellite loci for *O. brevirostris* in Thailand.

Table S2 - GenBank accession number of all the 32 mtDNA sequences including 15 haplotypes for *O. brevirostris* in Thailand.

Table S3 - Genetic diversity parameters in four sampling locations of *O. brevirostris* in Thailand.
