# Supplementary material for: Genetic structure of the endangered Irrawaddy dolphin (Orcaella brevirostris) in the Gulf of Thailand
Source: Genet Mol Biol. 2021 Apr 2;44(2):e20200365. doi: 10.1590/1678-4685-GMB-2020-0365 (PMC8022665; doi:10.1590/1678-4685-GMB-2020-0365)
Supplement: Table S1 - [file 1415-4757-GMB-44-2-e20200365-s1.pdf]

Genetics and Molecular Biology
https://doi.org/10.1590/1678-4685-GMB-2020-0365

Supplementary Material to "Genetic structure of the endangered Irrawaddy dolphin (*Orcaella brevirostris*) in the Gulf of Thailand"

Table S1 - Multiplex design information for all the tested 18 microsatellite loci for *O. brevirostris* in Thailand

Multiplex panels	Locus	Repeat motif	Primer sequences (5′-3′)	Primer dosage (μL)	annealing temperature (°C)	GenBank accession number	Fluorescent label
Multiplex 1	Sch5878	$(CAAC)_{12}$	F: TCTCCAGTGTTTGGGCTCTT	0.20	61	MK766860	6-FAM
			R: ACATTTTGGAAGGCAAGCTG	0.20			
	Sch6660	$(AAGG)_{13}$	F: CTGAGTGGTCCTCAAGGGAG	0.18		MK766861	VIC
			R: TCTGCTGACATGCCTCACTC	0.18			
	Sch443	$(CCAT)_{12}$	F: GGACTACAAGAAGCTGGGCA	0.18		MK766850	NED
			R: CTGGTGCGTGTAGCTGTTGT	0.18			
Multiplex 2	Sch10207	$(CATC)_{12}$	F: CCCTCTCTTGCTCTCTCCCT	0.15	62	MK766870	6-FAM
			R: TGTCTATTGTACAGCAGGATGGA	0.15			
	Sch843	$(AAAT)_{11}$	F: GAGAAACATTTTGTCTAAGTGCTCTG	0.15		MK766851	VIC
			R: GAACGCAGATCCTAACGTCTAATTAG	0.15			
	Sch7424	$(ATGG)_{13}$	F: GGAAGGGTGGATGGTTAGGT	0.15		MK766864	NED
			R: ATGTTCCCTGAGGATTGTGC	0.15			
Multiplex 3	Sch7357	$(ATGG)_{11}$	F: CAGTGCCTCGAACAGAGATTG	0.15	61	MK766863	6-FAM
			R: AAGTATTCCCACACCCATCCA	0.15			
	Sch193	$(AGAGA)_{12}$	F: GTATGGAAGGAAGGGAGGGA	0.20		MK766846	VIC
			R: CAAACTAAGGAAGCAAATGCAG	0.20			
	Sch8186	$(CCAT)_{11}$	F: CCCACAGAAGTCAAGCATCA	0.15		MK766865	NED
			R: CTGGAATCTGGGGTGAGAAAT	0.15			
Multiplex 4	Sch123	$(CCTAAC)_{7}$	F: GGAAGCCAGGTACCACTGTTG	0.15	63	MK766845	6-FAM
			R: TGAGGACAGCACAGACCAGAG	0.15			
	Sch4657	$(TTCC)_{11}$	F: TGTCCATGCAAGCGTAAATC	0.15		MK766855	VIC
			R: AGTGTTGGCATTTCTCCAGC	0.15			
	Sch5373	$(GATG)_{11}$	F: GGCTCCAGAGCTTGTGATCT	0.15		MK766858	NED
			R: GGAAGTCCATCTCCCTCTCC	0.15			

Multiplex panels	Locus	Repeat motif	Primer sequences (5′-3′)	Primer dosage (μL)	annealing temperature (℃)	GenBank accession number	Fluorescent label
Multiplex 5	Sch9144	$(ATCT)_{14}$	F: TAGAGCCTGCATGAGTGTGG	0.15	59	MK766868	6-FAM
			R: CAACAGAGTCAGCGTGCCT	0.15			
	Sch2513	$(CATC)_{13}$	F: GGGTTTACACCTGTCGCTGT	0.15		MK766854	VIC
			R: TCAACACATCATTGCCGAAT	0.15			
	Sch5685	$(AGGA)_{12}$	F: CATTCTTCCAGATGTACGTCCA	0.25		MK766859	NED
			R: CCTCGGGTAAGTCCCTCTTC	0.25			
Multiplex 6	Sch974	$(GTTTT)_{13}$	F: GCTGAGGATATCAGGGTGGA	0.15	59	MK766849	6-FAM
			R: CAGGGAAGTCCCAGAAATCA	0.15			
	Sch5094	$(TCTA)_{11}$	F: CTGGGGTTTCTAGCTTGCAG	0.15		MK766857	VIC
			R: ATTCTCCAGAGGAACCAGCA	0.15			
	Sch8947	$(CTAT)_{12}$	F: GGGAAAGATGCCAATCTGAA	0.25		MK766867	NED
			R: CGTACCGCAACAAAGAGTGA	0.25			
