# Supplementary material for: Genetic structure of the endangered Irrawaddy dolphin (Orcaella brevirostris) in the Gulf of Thailand
Source: Genet Mol Biol. 2021 Apr 2;44(2):e20200365. doi: 10.1590/1678-4685-GMB-2020-0365 (PMC8022665; doi:10.1590/1678-4685-GMB-2020-0365)
Supplement: Table S2 - [file 1415-4757-GMB-44-2-e20200365-s2.pdf]

Genetics and Molecular Biology
https://doi.org/10.1590/1678-4685-GMB-2020-0365

Supplementary Material to "Genetic structure of the endangered Irrawaddy dolphin (*Orcaella brevirostris*) in the Gulf of Thailand"

Table S2 - GenBank accession number of all the 32 mtDNA sequences including 15 haplotypes for *O. brevirostris* in Thailand

Haplotype	GenBank accession number
Hap-1	MT738330、MT738331、MT738337、MT738348
Hap-2	MT738349、MT738353、MT738354、MT738355、MT738357、MT738358
Hap-3	MT738356
Hap-4	MT738342、MT738345
Hap-5	MT738334、MT738335、MT73836
Hap-6	MT738338、MT738344
Hap-7	MT738361
Hap-8	MT738333
Hap-9	MT738350、MT738352
Hap-10	MT738359
Hap-11	MT738346
Hap-12	MT738360
Hap-13	MT738351
Hap-14	MT738339、MT738340、MT738341、MT738343、MT738347
Hap-15	MT738332
