# Supplementary material for: Genetic structure of the endangered Irrawaddy dolphin (Orcaella brevirostris) in the Gulf of Thailand
Source: Genet Mol Biol. 2021 Apr 2;44(2):e20200365. doi: 10.1590/1678-4685-GMB-2020-0365 (PMC8022665; doi:10.1590/1678-4685-GMB-2020-0365)
Supplement: Table S3 - [file 1415-4757-GMB-44-2-e20200365-s3.pdf]

Genetics and Molecular Biology
https://doi.org/10.1590/1678-4685-GMB-2020-0365

Supplementary Material to “Genetic structure of the endangered Irrawaddy dolphin (*Orcaella brevirostris*) in the Gulf of Thailand”

Table S3 - Genetic diversity parameters in four sampling locations of *O. brevirostris* in Thailand

Population	Locus	N	Na	Ne	I	Ho	He	uHe	F	P_{HWE}
**WG**	Sch6660	5	2	1.724	0.611	0.200	0.420	0.467	0.524	0.241
	Sch443	5	3	1.515	0.639	0.400	0.340	0.378	-0.176	0.958
	Sch10207	5	2	1.923	0.673	0.800	0.480	0.533	-0.667	0.136
	Sch843	5	5	3.571	1.418	1.000	0.720	0.800	-0.389	0.756
	Sch7357	5	2	2.000	0.693	0.600	0.500	0.556	-0.200	0.655
	Sch8186	5	4	2.381	1.089	0.800	0.580	0.644	-0.379	0.898
	Sch123	4	3	1.684	0.736	0.500	0.406	0.464	-0.231	0.931
	Sch9144	5	3	2.174	0.898	0.400	0.540	0.600	0.259	0.644
	Sch2513	5	3	1.515	0.639	0.400	0.340	0.378	-0.176	0.958
	Sch8947	5	3	1.515	0.639	0.200	0.340	0.378	0.412	0.019*
**AS**	Sch6660	2	2	1.600	0.562	0.500	0.375	0.500	-0.333	0.637
	Sch443	2	2	1.600	0.562	0.500	0.375	0.500	-0.333	0.637
	Sch10207	2	4	4.000	1.386	1.000	0.750	1.000	-0.333	0.423
	Sch843	2	2	1.600	0.562	0.500	0.375	0.500	-0.333	0.637
	Sch7357	2	2	1.600	0.562	0.500	0.375	0.500	-0.333	0.637
	Sch8186	2	2	2.000	0.693	0.000	0.500	0.667	1.000	0.157
	Sch123	2	3	2.667	1.040	1.000	0.625	0.833	-0.600	0.572
	Sch9144	1	2	2.000	0.693	1.000	0.500	1.000	-1.000	0.317
	Sch2513	2	3	2.667	1.040	1.000	0.625	0.833	-0.600	0.572
	Sch8947	2	1	1.000	0.000	0.000	0.000	0.000	ND	ND
**EG**	Sch6660	9	4	2.746	1.132	0.667	0.636	0.673	-0.049	0.640
	Sch443	9	3	2.160	0.854	0.667	0.537	0.569	-0.241	0.705
	Sch10207	9	2	1.528	0.530	0.222	0.346	0.366	0.357	0.284
	Sch843	9	5	4.154	1.509	0.778	0.759	0.804	-0.024	0.458
	Sch7357	9	2	2.000	0.693	0.556	0.500	0.529	-0.111	0.739
	Sch8186	9	4	1.421	0.634	0.333	0.296	0.314	-0.125	0.999
	Sch123	9	6	4.378	1.642	0.667	0.772	0.817	0.136	0.045*
	Sch9144	8	4	2.723	1.143	0.625	0.633	0.675	0.012	0.072
	Sch2513	9	5	1.841	0.961	0.333	0.457	0.484	0.270	0.487
	Sch8947	9	4	1.604	0.761	0.222	0.377	0.399	0.410	0.164
**NG**	Sch6660	12	3	1.524	0.616	0.333	0.344	0.359	0.030	0.148
	Sch443	12	5	3.032	1.311	0.750	0.670	0.699	-0.119	0.919
	Sch10207	12	2	1.385	0.451	0.333	0.278	0.290	-0.200	0.488

Population	Locus	N	Na	Ne	I	Ho	He	uHe	F	P_{HWE}
	Sch843	12	4	2.969	1.232	0.833	0.663	0.692	-0.257	0.249
	Sch7357	12	2	2.000	0.693	0.500	0.500	0.522	0.000	1.000
	Sch8186	12	3	1.524	0.616	0.250	0.344	0.359	0.273	0.565
	Sch123	11	6	3.507	1.453	0.636	0.715	0.749	0.110	0.545
	Sch9144	12	9	6.698	2.024	0.833	0.851	0.888	0.020	0.017*
	Sch2513	12	3	1.767	0.723	0.417	0.434	0.453	0.040	0.921
	Sch8947	12	3	1.405	0.544	0.333	0.288	0.301	-0.157	0.923

Note: N is the sample size, Na is the number of alleles, Ne is the number of effective alleles, I is the Shannon′s information index, Ho is the observed heterozygosity, He is the expected heterozygosity, uHe is the unbiased expected heterozygosity, F is the fixation index, P_{HWE} is the P value of Hardy-Weinberg equilibrium test, * represents $P < 0.05$ after Bonferroni corrections, ND represents not done.
